# Bioremediation Experience Collected in “Bioengineering in Remediation of Polluted Environments”: A Closing Perspective by Guest Editors

**DOI:** 10.3390/bioengineering12020122

**Published:** 2025-01-28

**Authors:** Marco Zeppilli, Bruna Matturro

**Affiliations:** 1 Department of Chemistry, University of Rome Sapienza, Piazzale Aldo Moro 5, 00185 Rome, Italy; 2 Research Center for Applied Sciences to the Safeguard of Environment and Cultural Heritage (CIABC), University of Rome Sapienza, Piazzale Aldo Moro 5, 00185 Rome, Italy; 3 Water Research Institute (IRSA), National Research Council (CNR), 00010 Montelibretti, Italy; 4 National Biodiversity Future Center, 90133 Palermo, Italy

**Keywords:** bioremediation, resource recovery, pollutant removal

The article collection entitled “*Bioengineering in Remediation of Polluted Environments*” was launched in September 2021. Its main scope was innovative contributions related to the bioremediation field of contaminated environmental matrixes. Indeed, contamination currently affects several aspects of environmental and human health due to the wide spread of target molecules produced by human activities [1].

Anthropogenic activities have significantly impacted environmental quality, leading to the release of toxic organic and inorganic compounds into soil, water, and sediments. Over the past 30 years, substantial research has been dedicated to developing sustainable strategies for mitigating and addressing contamination. Among these, environmental biotechnology—particularly bioremediation approaches—has emerged as a cost-effective and environmentally friendly solution [2]. Bioremediation restores contaminated matrices by mimicking and harnessing the self-restoration capacity inherent in living ecosystems. This approach involves stimulating and monitoring naturally occurring processes that restore polluted environments to their original state [3]. By leveraging the biological activities of microorganisms, plants, or enzymes, bioremediation aims to degrade, detoxify, or transform pollutants, making it a sustainable and eco-friendly technology for environmental cleanup. As mentioned, bioremediation technologies are considered the most promising approaches in terms of environmental sustainability [4,5].

The article collection “*Bioengineering in Remediation of Polluted Environments*” presents innovative contributions addressing some highly challenging environmental issues, focusing on sustainable methods for pollution mitigation, resource recovery, and waste management. Within this collection, nine contributions from various research groups were received. Following the review process, eight were accepted for publication, including four review papers and four research articles. 

In detail, the studies published address diverse topics ranging from photodegradation and bioremediation strategies to bioenergy recovery and sustainable plastic management, reflecting a broad spectrum of cutting-edge research aiming at fostering environmental resilience.

Together, these studies demonstrate the significant progress in developing sustainable technologies for environmental remediation and resource recovery. They collectively highlight the importance of integrating biological, chemical, and engineering solutions to tackle pollution, reduce waste, and create value from industrial by-products, by promoting circular economy principles and leveraging advanced biotechnological approaches. Review papers mostly included systematic and critical reviews of the most recent studies on biotechnology applications in the bioremediation of polluted matrixes, including groundwater, wastewater, and sludge treatment.

The review paper by Johnston and collaborators [6] presents a detailed literature review about the bioconversion of plastic waste into biodegradable plastics like polyhydroxyalkanoates (PHAs), exploiting microbial cultivation and oxidative degradation pathways of conventional plastics like polyethylene (PE), polypropylene (PP), polystyrene (PS), and poly(ethylene terephthalate) (PET). The review details the key advantages and ongoing challenges in optimizing microbial strains and pathways, describing possible routes towards circular carbon recovery and utilization from plastic pollution. Similarly, the review paper by Rodríguez-Fonseca and collaborators [7] focuses on the potential of *Streptomyces* strains in degrading synthetic polymers. Indeed, *Streptomyces* strains are well known for breaking down natural macromolecules like lignin and chitin, and in recent studies, they have shown limited but promising potential for biodegrading synthetic plastics such as polyethylene. Although research in this area is in its early stages, exploring *Streptomyces* strains’ diverse ecological niches may uncover new applications for addressing the persistent challenge of plastic waste. The third review paper [8] published in the article collection focused on a common class of groundwater contaminants named chlorinated aliphatic hydrocarbons (CAHs), which represent a ubiquitous contaminant of former industrial areas worldwide. The review paper by Rossi and collaborators [8] focuses on different strategies for biological reductive dechlorination stimulation for CAH removal in groundwater. It analyzes the recent literature about CAH bioremediation, combining bio-based materials like polyhydroxyalkanoates and biochar with novel bio-electrochemical systems. Additionally, the contamination of soil matrixes by pesticides was analyzed and described in the fourth review paper on pollution in agricultural soils. The review by Raffa and Chiampo [9] presents the utilization of bacterial, fungal, or enzymatic processes as a cost-effective and environmentally friendly solution to degrade pesticide residues. The review paper describes the efficiency of these processes depending on the pollutant type and soil conditions, as well as regulatory frameworks governing pesticide use.

Research articles contained in the article collection covered different experimental and modeling approaches for the utilization of bio-based methods to remove target compounds. The experimental study by Huang and collaborators [10] focuses on using hypocrellins (HYPs) from *Ascomycota fungi* for the photocatalytic degradation of Rhodamine B (RhB), a common organic pollutant usually present in industrial wastewater streams, especially in effluent streams from printing and dyeing. HYPs exhibit pseudo-first-order kinetics related to the combination of hydrogen peroxide (H_2_O_2_) under visible light, achieving an 82.4% degradation of RhB within 60 min. Moreover, the experimental study showed the self-degradability of HYPs under alkaline conditions, preventing the risk of secondary pollution. With a similar biophotocatalytic approach, the experimental study by Tetteh and Rathilal [11] investigates an engineered Fe-TiO_2_ catalyst for chemical oxygen demand (COD) removal and biogas production from industrial wastewater. The experimental study, which investigates the effects of catalyst load and hydraulic retention time, conducted modeling based on response surface methodology (RSM) to optimize process parameters to increase biogas production and COD removal.

The third research article, published by Charria-Girón and collaborators [12], focuses on microalgae cultivation as a strategy for valorizing industrial by-products. The study focuses on the valorization of an industrial anaerobic sludge, coming from the biological treatment of a baker’s yeast company, as a potential source of nutrients. Indeed, industrial anaerobic sludge, pretreated to extract nutrients, is an effective medium for *Chlorella sorokiniana* cultivation, enabling the cultivation of nitrogen-deprived microalgae to enhance both biomass formation and fatty acid synthesis.

The fourth research paper explored the potential of nitrogen-fixing bacteria (NFB) in biofertilization. The study published by Rodriguez-Gonzalez and collaborators [13] performs tests in extreme conditions. The microbial communities within the sludge demonstrate resilience under extreme conditions, as highlighted in another study examining aerobic sludges enriched with NFB to provide a sustainable alternative to chemical fertilizers. These sludges, resistant to high concentrations of ammonium salts, retain robust nitrogen-fixing populations, including species such as *Fluviicola*, *Azospirillum*, and others, suggesting their utility in promoting nitrogen cycling and soil health. This eco-friendly approach not only reduces dependence on chemical fertilizers but also mitigates environmental harm associated with their overuse.

The article collection disseminated different studies concerning the utilization of bio-based solutions for environmental issues. It received 181 citations and significantly contributed to the first impact factor received by the *Bioengineering* journal in 2022.

Throughout the article collection, bioremediation strategies showed their potential as part of hybrid technologies involving external energy inputs such as sunlight energy or bio-electrochemical systems for reducing power supply in chlorinated aliphatic hydrocarbon removal. The development of hybrid techniques is a win–win strategy in the field of process development; indeed, the combination of chemical, physical, electrochemical, and bio-based solutions can overcome the target limitations of the different processes. As an example, in the studies conducted by Huang and collaborators [5] and Tetteh and Rathilal [6], biomolecules produced by selected microorganisms interacted with sunlight captured by engineered inorganic materials (e.g., Fe-TiO_2_ catalyst). The article collection also highlighted the importance of the scientific investigation of living microorganisms’ different metabolic pathways to better understand their potential role in developing more sustainable solutions for the remediation of environmental matrixes. Microorganisms including fungi, bacteria, and microalgae were investigated for different purposes under different conditions to achieve the removal of target compounds or valorize wastewater through bioenergy or target bioproduct production.
